# Alumina‐Supported Alpha‐Iron(III) Oxyhydroxide as a Recyclable Solid Catalyst for CO_2_ Photoreduction under Visible Light

**DOI:** 10.1002/anie.202204948

**Published:** 2022-05-12

**Authors:** Daehyeon An, Shunta Nishioka, Shuhei Yasuda, Tomoki Kanazawa, Yoshinobu Kamakura, Toshiyuki Yokoi, Shunsuke Nozawa, Kazuhiko Maeda

**Affiliations:** ^1^ Department of Chemistry School of Science Tokyo Institute of Technology 2-12-1-NE-2 Ookayama, Meguro-ku Tokyo 152-8550 Japan; ^2^ Nanospace Catalysis Unit Institute of Innovative Research Tokyo Institute of Technology 4259 Nagatsuta-cho, Midori-ku Yokohama 226-8503 Japan; ^3^ Institute of Materials Structure Science High Energy Accelerator Research Organization 1-1 Oho, Tsukuba Ibaraki 305-0801 Japan; ^4^ Japan Society for the Promotion of Science Kojimachi Business Center Building 5-3-1 Kojimachi, Chiyoda-ku Tokyo 102-0083 Japan

**Keywords:** Artificial Photosynthesis, Earth-Abundant Metals, Iron, Photocatalysis, Solar Fuels

## Abstract

Photocatalytic conversion of CO_2_ into transportable fuels such as formic acid (HCOOH) under sunlight is an attractive solution to the shortage of energy and carbon resources as well as to the increase in Earth's atmospheric CO_2_ concentration. The use of abundant elements as the components of a photocatalytic CO_2_ reduction system is important, and a solid catalyst that is active, recyclable, nontoxic, and inexpensive is strongly demanded. Here, we show that a widespread soil mineral, alpha‐iron(III) oxyhydroxide (α‐FeOOH; goethite), loaded onto an Al_2_O_3_ support, functions as a recyclable catalyst for a photocatalytic CO_2_ reduction system under visible light (*λ*>400 nm) in the presence of a Ru^II^ photosensitizer and an electron donor. This system gave HCOOH as the main product with 80–90 % selectivity and an apparent quantum yield of 4.3 % at 460 nm, as confirmed by isotope tracer experiments with ^13^CO_2_. The present work shows that the use of a proper support material is another method of catalyst activation toward the selective reduction of CO_2_.

## Graphical abstract

A widespread soil mineral, alpha‐iron(III) oxyhydroxide, which is loaded onto an alumina support, works as a recyclable catalyst for CO_2_ reduction. In the presence of a Ru^II^ photosensitizer, the catalyst produces formic acid with 80–90 % selectivity and an apparent quantum yield of 4.3 % at 460 nm.

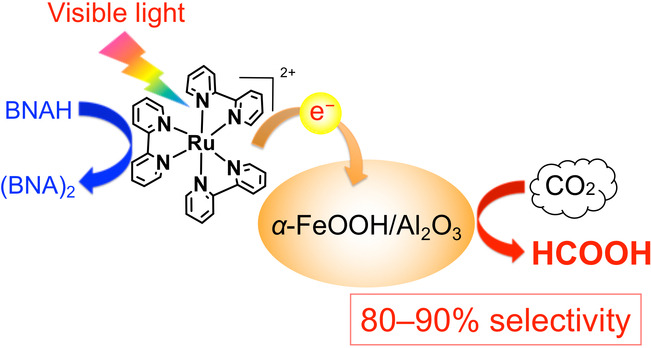

In recent years, CO_2_ emissions arising from the consumption of fossil fuels has become a serious problem. Among various proposed methods and schemes to address this problem, photocatalytic CO_2_ reduction is expected to be a key technology in the future. The production of transportable fuels such as formic acid (HCOOH) has attracted attention because they can act as energy carriers of hydrogen, which releases a high density of energy after combustion, without generating byproducts other than water.[^1^, ^2^]

A photocatalytic CO_2_ reduction system typically consists of a light‐absorbing substrate (e.g., a molecular redox photosensitizer or semiconductor) and a catalyst (Scheme 1).[^3^, ^4^, ^5^] Because the reduction of CO_2_ into value‐added chemicals involves the transfer of at least two electrons, a catalyst that enables multi‐electron transfer is necessary. Thus far, various metal complexes based on abundant metals such as Mn^I^, Fe^II^, Co^I^, and Ni^II^ have been reported as efficient catalysts for photocatalytic CO_2_ reduction.[^4^, ^6^]

**Scheme 1:**
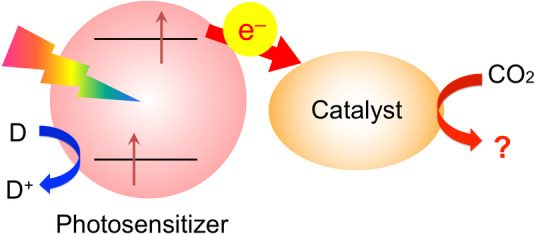
A photochemical CO_2_ reduction system consisting of a catalyst and a photosensitizer. D indicates an electron donor. The catalyst may be in the form of a molecule or nanoparticle.

The potential recyclability of solid catalysts distinguishes them from homogeneous molecular catalysts, and the development of a catalyst that is sufficiently active, nontoxic, and inexpensive is an important subject in this research field. Recently, solid materials such as ZnCo_2_O_4_,^[7]^ NiCo_2_O_4_,^[8]^ Co_3_O_4_,^[9]^ CoSn(OH)_6_,^[10]^ LaCoO_3_,^[11]^ and metal–organic frameworks (MOFs) containing Co[^12^, ^13^, ^14^] or Ni[^15^, ^16^] have been reported to catalyze CO_2_ reduction in the presence of a Ru^II^ photosensitizer and an electron donor under visible light.

For future practical applications, Fe‐based solid catalysts have advantages over catalysts based on other early transition metals such as Co and Ni. Some Fe‐containing solids have recently been reported to function as CO_2_ reduction catalysts with [Ru(bpy)_3_]^2+^. For example, a composite derived from a Ni‐ and Fe‐containing MOF, further combined with SnO_2_, promoted the reduction of CO_2_ to CO.^[17]^ ZnFe_2_O_4_ nanoparticles grown in situ on the surface of iron porphyrin covalent triazine‐based frameworks^[18]^ and Co_3_O_4_/CoFe_2_O_4_ nanoparticles^[19]^ have been reported to exhibit a similar functionality, although the carbon source of the reaction product has not been fully clarified in these works because of the lack of ^13^CO_2_ experiments, which is one of the most important aspects in photochemical CO_2_ reduction studies (especially in heterogeneous systems).^[20]^ MOFs containing Fe^2+^ and another metal cation (e.g., Mn, Co, Ni, or Zn) have been reported to function as catalysts for CO_2_ reduction with [Ru(bpy)_3_]^2+^, giving CO as the main product with 75–85 % selectivity, as confirmed by a tracer experiment with ^13^CO_2_.^[21]^ As such, Fe‐based catalysts reported thus far produce CO as the main product; in addition, HCOOH‐generating Fe‐based solid catalysts for photochemical CO_2_ reduction have rarely been reported.

Here, we report that α‐FeOOH‐loaded Al_2_O_3_ can function as a recyclable catalyst for visible‐light‐driven CO_2_ reduction in the presence of a Ru^II^ photosensitizer ([Ru(bpy)_3_]^2+^, abbreviated as **Ru**]) and 1‐benzyl‐1,4‐dihydronicotinamide (BNAH) as an electron donor. Under these conditions, photoexcited **Ru** undergoes reductive quenching by reaction with BNAH,^[22]^ which was also confirmed in the present study (see Figure S1 and additional discussion). Therefore, CO_2_ reduction proceeds if a proper catalyst is present in the reaction system. In fact, the α‐FeOOH/Al_2_O_3_ catalyst produces HCOOH as the main product via CO_2_ reduction, with 80–90 % selectivity.

A catalyst sample, Fe‐loaded Al_2_O_3_, was prepared by a simple impregnation and H_2_‐reduction method. As evident in Figure 1a, the Al_2_O_3_ support was composed of well‐crystallized ≈1 μm particles, which form larger secondary particles with some interparticle space. Upon loading of Fe, the “interconnection” of particles was more pronounced and the interparticle space was reduced, accompanied by some surface roughening (see additional images in Figure S2). Energy‐dispersive X‐ray spectroscopy (EDS) measurements revealed that Fe species were well distributed on the Al_2_O_3_ surface (Figure 1b). The X‐ray diffraction (XRD) pattern of the Fe‐loaded Al_2_O_3_ was recorded to investigate the crystal structure of the Fe species in the sample. However, no diffraction peak other than those of Al_2_O_3_ was observed in the pattern of the Fe‐loaded Al_2_O_3_ (Figure S3), suggesting that the Fe species loaded onto Al_2_O_3_ was amorphous and/or in the form of a thin layer below the diffraction limit.

**Figure 1:**
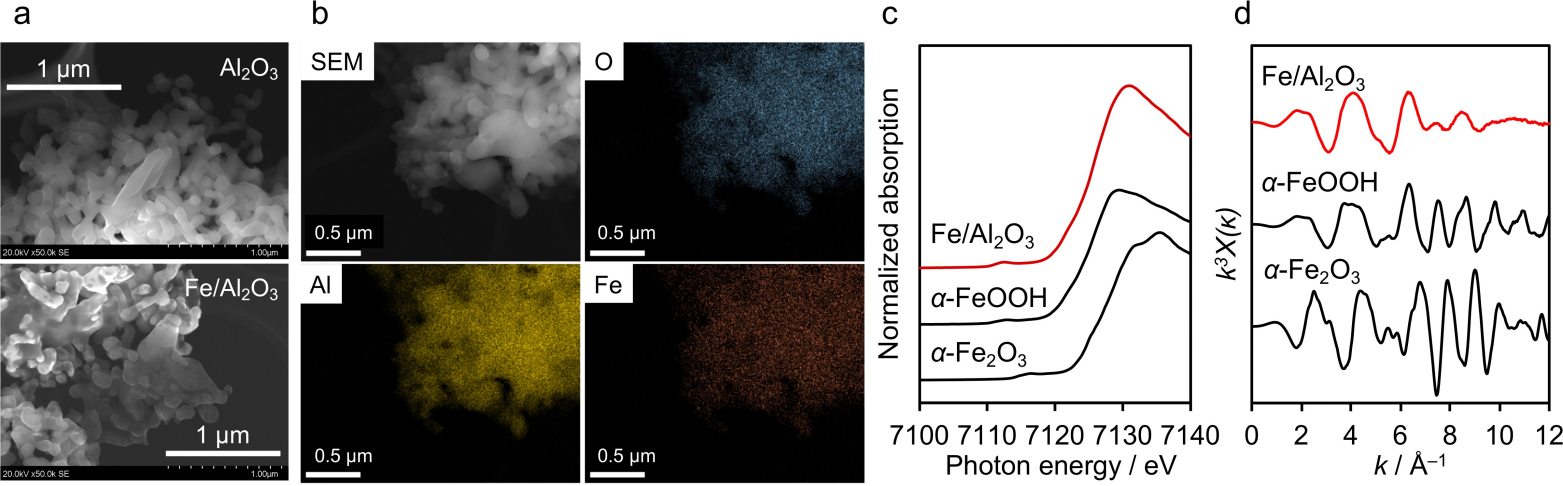
Characterization of Fe‐loaded Al_2_O_3_: a) SEM images and b) EDS mapping images. Fe K‐edge c) XANES spectra and d) EXAFS oscillation.

To identify the local structure of Fe species loaded onto Al_2_O_3_, X‐ray absorption fine structure (XAFS) measurements were conducted. Figure 1c shows the Fe K‐edge X‐ray absorption near edge structure (XANES) spectrum of the Fe‐loaded Al_2_O_3_, along with the spectra of α‐Fe_2_O_3_ and α‐FeOOH for reference. From the characteristic feature of the pre‐edge region (7113 eV), the Fe species loaded onto Al_2_O_3_ were found to be very similar to α‐FeOOH but different from α‐Fe_2_O_3_. FeOOH has several stable polymorphs, and α‐FeOOH is one of the most stable phases.^[23]^ In the present work, the existence of α‐FeOOH on the Al_2_O_3_ surface was also supported by the extended X‐ray absorption fine structure (EXAFS) oscillation. As shown in Figure 1d, the EXAFS oscillation of the Fe‐loaded Al_2_O_3_ was similar to that of the α‐FeOOH reference, although the oscillation was relatively weak at larger *k* regions, most likely because of the thin‐layer form of the loaded Fe species on the Al_2_O_3_.^[24]^ The existence of α‐FeOOH on Al_2_O_3_ was also supported by the result of X‐ray photoelectron spectroscopy (Figure S4). On the basis of these results, the prepared sample is hereafter represented as α‐FeOOH/Al_2_O_3_.

Photocatalytic CO_2_ reduction was performed at room temperature using α‐FeOOH/Al_2_O_3_ in an *N*,*N*‐dimethylacetamide (DMA)/BNAH mixed solution in the presence of **Ru** under visible light (*λ*>400 nm). As listed in Table 1, the α‐FeOOH/Al_2_O_3_ gave HCOOH as the main product; CO and H_2_ were also produced as secondary products. The CO_2_ reduction selectivity to HCOOH was 82 %. The apparent quantum yield (AQY) for HCOOH formation was 4.3 % at 460 nm. No reaction occurred in the dark or in the absence of α‐FeOOH (only Al_2_O_3_) (entries 2 and 3). No CO_2_ reduction product was obtained in the absence of either **Ru**, CO_2_, or BNAH (entries 4–6). As shown in Figure S5, the α‐FeOOH/Al_2_O_3_ showed semiconductor‐like absorption in the visible‐light region; however, this absorption was not responsible for the visible‐light CO_2_ reduction. Notably, **Ru** can undergo structural transformation during photochemical reaction to become catalytically active species for HCOOH production.[^25^, ^26^] However, we confirmed that HCOOH production in the “blank” system was negligible under the present reaction conditions (entry 7).

**Table 1:**
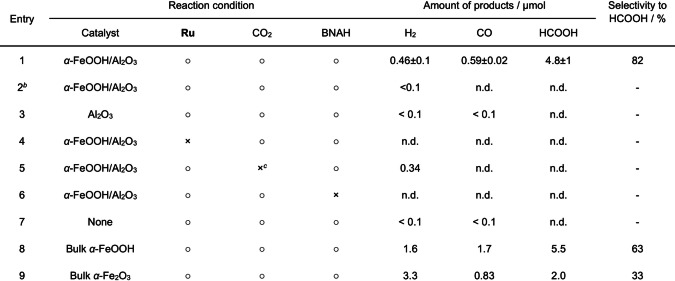
Results of visible‐light CO_2_ reduction experiments (*λ*>400 nm).^[a]^

[a] Reaction conditions: catalyst, 4 mg (Fe loaded 10.0 wt % to catalyst); solution, 4 mL DMA containing 1.0 mM **Ru** and 0.1 M BNAH; reaction time, 3 h. [b] In the dark. [c] Under an Ar atmosphere. n.d.=Not detected.

Figure 2a shows a typical time course of CO_2_ reduction using the α‐FeOOH/Al_2_O_3_ catalyst. The amount of HCOOH produced increased with reaction time, but changed little after 3 h. One of the most probable reasons for the deactivation is the decomposition and/or structural change of [Ru(bpy)_3_]^2+^ during the reaction (see Figure S6). Nevertheless, the performance of the α‐FeOOH/Al_2_O_3_ catalyst was found to be stable in consecutive runs without loss of the high selectivity to HCOOH (85–90 %), as shown in Figure 2b. The Fe‐based turnover number (TON) exceeded 6 after the five consecutive runs, confirming the catalytic cycle of the reaction. It was also confirmed by isotope tracer experiments with ^13^CO_2_ that both HCOOH and CO originated from CO_2_ introduced into the reaction system (see Figure S7).

**Figure 2:**
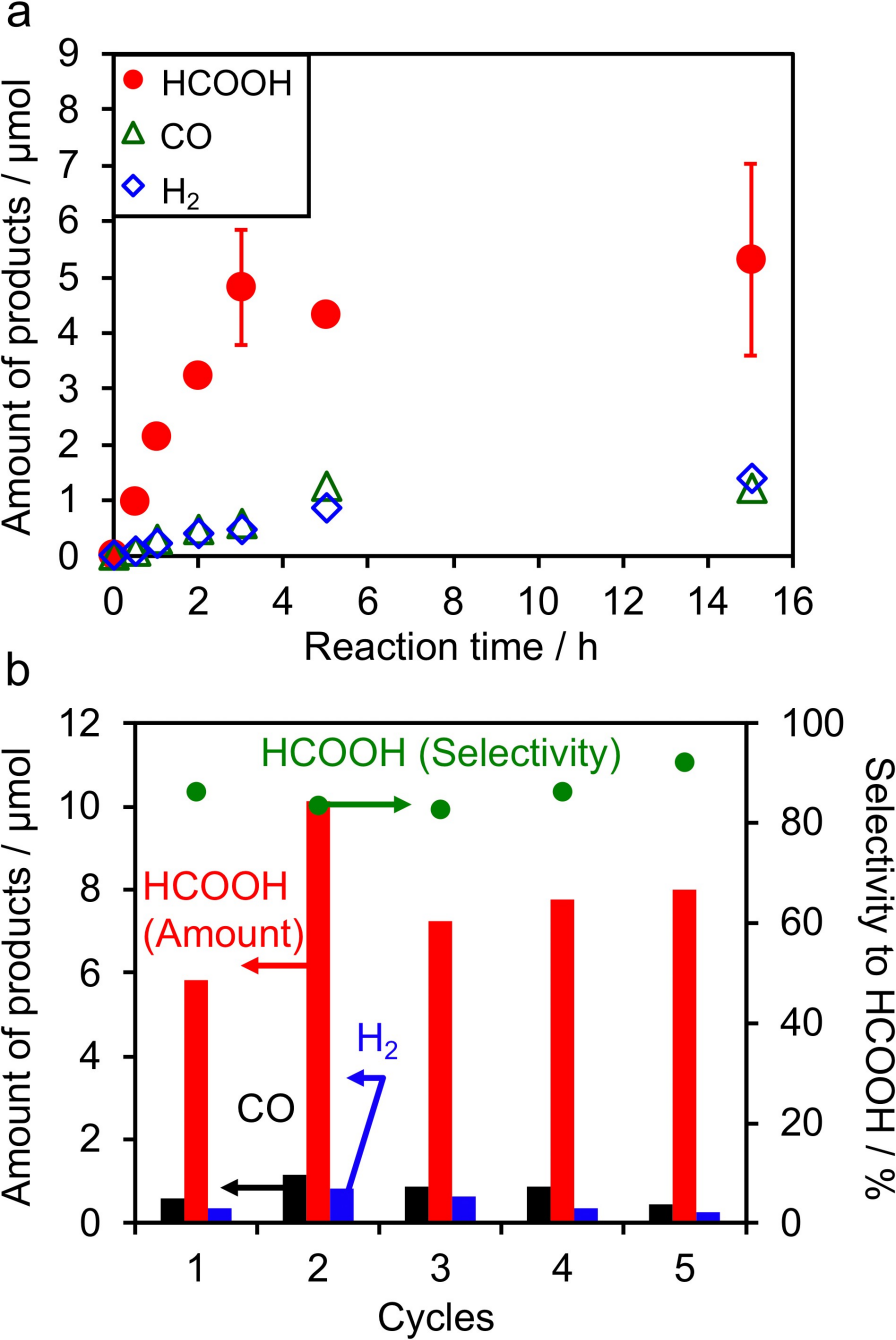
a) A typical time course of CO_2_ reduction using α‐FeOOH/Al_2_O_3_ under visible light (*λ*>400 nm). Reaction conditions: catalyst, 4 mg (Fe loaded 10.0 wt % to catalyst); solution, 4 mL DMA containing 1.0 mM **Ru** and 0.1 M BNAH. b) Amounts of reaction products and the HCOOH selectivity in photocatalytic CO_2_ reduction using α‐FeOOH/Al_2_O_3_ under visible light (*λ*>400 nm). Each run was conducted for 3 h, and the α‐FeOOH/Al_2_O_3_ catalyst was subsequently recovered by centrifugation. The next reaction was then started using a new reaction solution and the recovered catalyst.

The CO_2_ reduction performance was also dependent on the loading amount of α‐FeOOH on Al_2_O_3_. As shown in Figure S8, the performance improved as the α‐FeOOH loading amount was increased to 10.0 wt %; at greater loading amounts, the performance was adversely affected. A commercially available bulk α‐FeOOH achieved CO_2_ reduction to HCOOH at a rate comparable to that of the α‐FeOOH/Al_2_O_3_ catalyst; however, the selectivity to HCOOH was lower (entry 8). These results imply that highly dispersed α‐FeOOH is important for selective CO_2_ reduction. A well‐known iron(III) oxide, α‐Fe_2_O_3_, also functioned as a catalyst but exhibited lower CO_2_ reduction performance (entry 9).

As mentioned earlier, it is most likely that the present CO_2_ photoreduction system with the [Ru(bpy)_3_]^2+^ photosensitizer works according to a reductive quenching mechanism, in which one‐electron‐reduced species of the Ru complex donates an electron to solid catalysts and the concomitant CO_2_ reduction occurs. It is also noted that the α‐FeOOH/Al_2_O_3_ catalyst produced H_2_ in the absence of CO_2_ (Table 1, entry 5). A similar result has been reported when a molecular catalyst was employed instead of the solid catalyst under the condition identical to the present.^[22]^ Therefore, reduction of CO_2_ and proton can compete with each other, depending on the environment of the catalyst surface as well as CO_2_ adsorption capability of the catalyst.

CO_2_ adsorption capabilities of the catalyst samples were examined. A shown in Figure 3, the bulk α‐FeOOH had the ability to adsorb CO_2_, consistent with early reports,[^27^, ^28^] which was superior to α‐Fe_2_O_3_. More importantly, the α‐FeOOH/Al_2_O_3_ catalysts captured CO_2_ more efficiently than the bulk α‐FeOOH. It is also noted that catalysts having higher CO_2_ adsorption capability tended to give higher CO_2_ reduction activity and selectivity. However, this was not the case for the 30.0 wt % sample, which had a little higher CO_2_ adsorption capability, but lower CO_2_ reduction activity than the optimal 10.0 wt % sample (Figure S8). In the 30.0 wt % sample, aggregation of the loaded Fe species was observed (Figure S9), and the lower activity is in line with a general trend in heterogeneous catalysis; i.e., aggregated species give lower catalytic activity. Nevertheless, the 30.0 wt % sample still maintained its high CO_2_ reduction selectivity. Considering these results, the primary role of the Al_2_O_3_ support is concluded to provide α‐FeOOH with suitable dispersion, thereby improving the adsorption of CO_2_, which is essential to increasing the CO_2_ reduction activity and selectivity.

**Figure 3:**
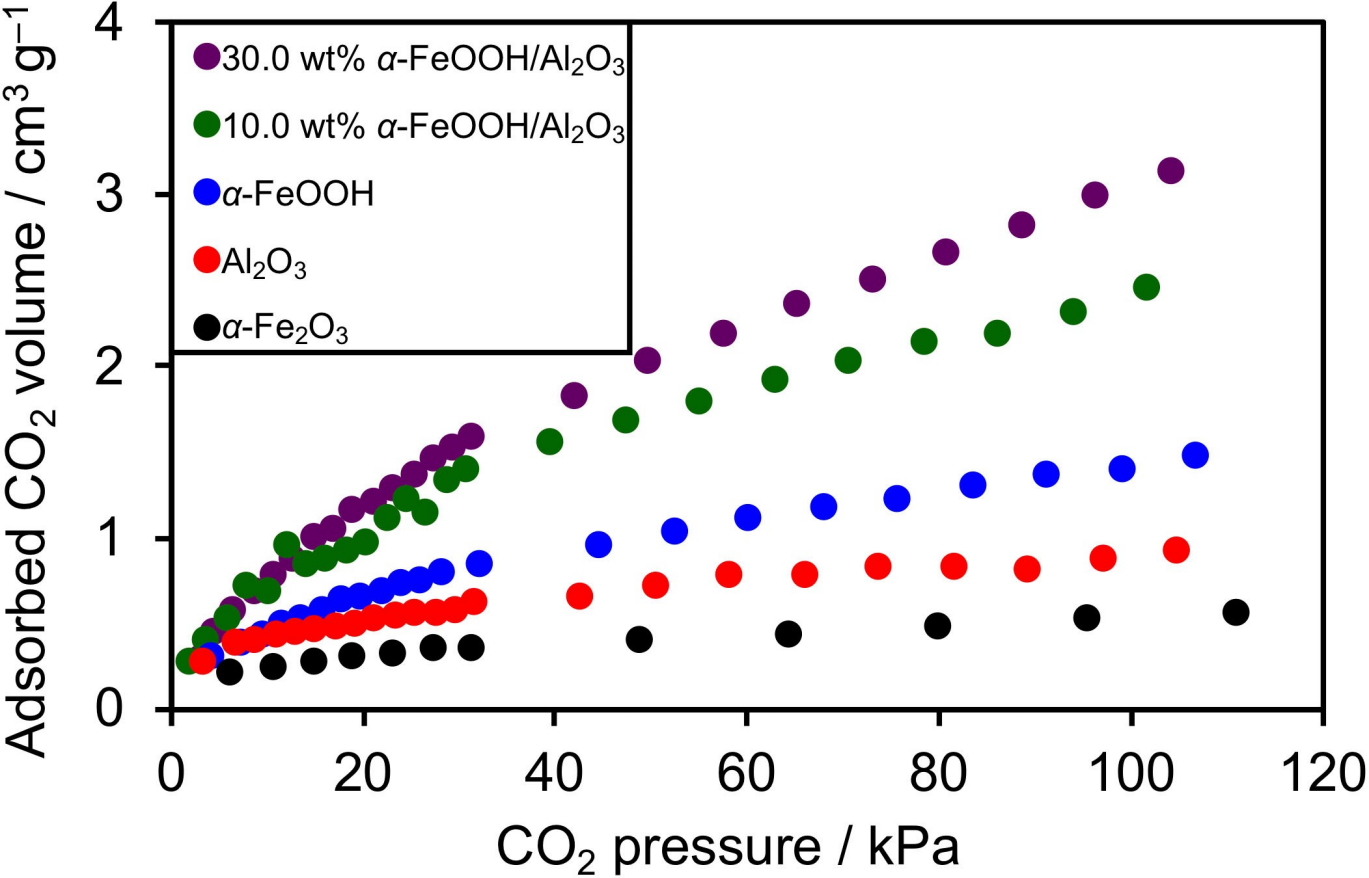
CO_2_ sorption isotherms of catalyst samples at 298 K.

As a homogeneous photocatalytic system for selective visible‐light CO_2_ reduction with the use of [Ru(bpy)_3_]^2+^ and BNAH as a photosensitizer and an electron donor, a molecular catalyst of [Ru(bpy)_2_(CO)_2_](PF_6_)_2_ has been reported by Ishida et al.^[22]^ According to that report, the homogeneous system produced nearly 1 : 1 HCOOH and CO from a DMA/BNAH mixed solution, with the total quantum yield for CO_2_ reduction being estimated to be ≈8.9 % at 460 nm. Therefore, the AQY of our system (4.3 %) was roughly half that of the representative homogeneous system. Considering the nature of heterogeneous catalysis where the reaction rate is generally slower compared to a homogeneous system, nevertheless, the present result would be a good starting point of further research on Fe‐based solid catalyst for efficient CO_2_ reduction.

In conclusion, we demonstrated that α‐FeOOH/Al_2_O_3_ functions as a recyclable catalyst for CO_2_ reduction to HCOOH with ≈90 % selectivity in the presence of [Ru(bpy)_3_]^2+^ and BNAH as a photosensitizer and an electron donor, respectively, under visible light. This catalyst is the first example of an Fe‐based solid catalyst for HCOOH generation that can function in a photochemical CO_2_ reduction scheme with the aid of a proper redox photosensitizer. More importantly, the results of this work suggest that, with the use of a proper support material (here, Al_2_O_3_), well‐known, earth‐abundant compounds can be used as selective catalysts for CO_2_ reduction without a complicated catalyst preparation method. Such a support effect has not been explored sufficiently in the search for solid catalysts applicable to photochemical CO_2_ reduction schemes. Therefore, much room exists for developing precious‐metal‐free, abundant compounds—which are, of course, not limited to α‐FeOOH—for reducing CO_2_ to energy‐rich chemicals, although mechanistic studies will be important.

## Conflict of interest

The authors declare no competing financial interest.

## Supporting information

As a service to our authors and readers, this journal provides supporting information supplied by the authors. Such materials are peer reviewed and may be re‐organized for online delivery, but are not copy‐edited or typeset. Technical support issues arising from supporting information (other than missing files) should be addressed to the authors.

Supporting Information

## Data Availability

The data that support the findings of this study are available from the corresponding author upon reasonable request.
