# Supplementary material for: Alumina‐Supported Alpha‐Iron(III) Oxyhydroxide as a Recyclable Solid Catalyst for CO2 Photoreduction under Visible Light
Source: Angew Chem Int Ed Engl. 2022 May 12;61(26):e202204948. doi: 10.1002/anie.202204948 (PMC9325401; doi:10.1002/anie.202204948)
Supplement: Supplementary file 1 — Supporting Information [file ANIE-61-0-s001.pdf]

Angewandte Chemie
Eine Zeitschrift der Gesellschaft Deutscher Chemiker

Supporting Information

Alumina-Supported Alpha-Iron(III) Oxyhydroxide as a Recyclable Solid Catalyst for CO_2 Photoreduction under Visible Light

*D. An, S. Nishioka, S. Yasuda, T. Kanazawa, Y. Kamakura, T. Yokoi, S. Nozawa, K. Maeda**

Supporting Information
©Wiley-VCH 2022
69451 Weinheim, Germany

Alumina-Supported Alpha-Iron(III) Oxyhydroxide as a Recyclable Solid Catalyst for CO_2 Photoreduction under Visible Light

Daehyeon An, Shunta Nishioka, Shuhei Yasuda, Tomoki Kanazawa, Yoshinobu Kamakura, Toshiyuki Yokoi, Shunsuke Nozawa, and Kazuhiko Maeda*

Abstract: Photocatalytic conversion of CO_2 into transportable fuels such as formic acid (HCOOH) under sunlight is an attractive solution to the shortage of energy and carbon resources as well as to the increase in Earth's atmospheric CO_2 concentration. The use of abundant elements as the components of a photocatalytic CO_2 reduction system is important, and a solid catalyst that is active, recyclable, nontoxic, and inexpensive is strongly demanded. Here, we show that a widespread soil mineral, alpha-iron(III) oxyhydroxide (α-FeOOH; goethite), loaded onto an Al_2O_3 support, functions as a recyclable catalyst for a photocatalytic CO_2 reduction system under visible light (λ > 400 nm) in the presence of a Ru(II) photosensitizer and an electron donor. This system gave HCOOH as the main product with 80–90% selectivity and an apparent quantum yield of 4.3% at 460 nm, as confirmed by isotope tracer experiments with $^{13}CO_2$. The present work shows that the use of a proper support material is another method of catalyst activation toward the selective reduction of CO_2.

DOI: 10.1002/anie.202204948

Table of Contents

Experimental procedures, pp. 3–4
- Materials and Reagents, p. 3
- Preparation of FeOOH/Al_2O_3, p. 3
- Characterization, p. 3
- Photocatalytic Reactions, p. 3
- Isotope Tracer Experiment, p. 4

Result and Discussion, pp. 5–9
- Figure S1. Steady-state emission spectra of $[Ru(bpy)_3]^{2+}$ in DMA solution containing catalysts and/or BNAH, p. 5
- Figure S2. SEM images of Fe-loaded Al_2O_3 and unloaded Al_2O_3, p. 5
- Figure S3. XRD patterns of Fe-loaded Al_2O_3, unloaded Al_2O_3, and the commercial α-FeOOH, p. 6
- Figure S4. Fe 2p and Al 2s XPS spectra of 10.0 wt% Fe-loaded Al_2O_3, p. 6
- Figure S5. UV–visible absorption spectra of $[Ru(bpy)_3]Cl_2 \cdot 6H_2O$ in DMA and the 10.0 wt% α-FeOOH/Al_2O_3, p. 7
- Figure S6. UV-visible absorption spectrum of the reaction solution after CO_2 reduction, p. 7
- Figure S7. Results of isotope tracer experiments with $^{13}CO_2$, p. 8
- Figure S8. Dependence of HCOOH generation over α-FeOOH/Al_2O_3 on the loading amount of Fe, p. 8
- Figure S9. SEM images of Fe-loaded Al_2O_3 catalysts with different Fe loadings, p. 8
- Additional note on the result of AQY measurement, p. 9

References, p. 9

Author contributions, p. 9

Experimental Section

Materials and Reagents. 1-Benzyl-1,4-dihydronicotinamide (BNAH) was prepared according to a method reported in the literature[1] and subsequently stored in a refrigerator under an Ar atmosphere. *N*,*N*-Dimethylacetamide (DMA; >99.0%, Kanto Chemical) was used after distillation. Al_2O_3 (AEROXIDE® Alu C) was purchased from NIPPON AEROSIL. $Ru(bpy)_3Cl_2·6H_2O$ (Sigma-Aldrich), $Fe(NO_3)_3·9H_2O$ (>99.0%, Kanto Chemical), NaOH (>95.0%, Kanto Chemical), HCOOH (>98%, Kanto Chemical), *α*-FeOOH (>95.0%, Kanto Chemical), *α*-Fe_2O_3 (99.9+%, Wako Pure Chemical Industries), and D_2O (99.9+%, Wako Pure Chemical Industries) were used as supplied. The specific surface areas of the *α*-FeOOH and *α*-Fe_2O_3 were 10.9 and 6.0 $m^2\ g^{-1}$, respectively.

Preparation of FeOOH/Al_2O_3. Prior to use, Al_2O_3 was calcined at 1573 K for 1 h in air. The specific surface area of the treated Al_2O_3 was 13 $m^2\ g^{-1}$. The Al_2O_3 powder was dispersed in 2 mL of water containing an appropriate amount of $Fe(NO_3)_3·9H_2O$ in an evaporating dish. The resultant suspension was stirred using a glass rod until the water was completely evaporated. The obtained powder was collected and heated in a H_2 stream (20 mL min^{-1}) at 473 K for 1 h. The loading amount of Fe was 10.0 wt% unless otherwise stated. In addition to Al_2O_3, other oxides were also tested as supports.

Characterization. X-ray diffraction (XRD) patterns were acquired using a Rigaku MiniFlex600 powder diffractometer equipped with a monochromatic Cu K*α* radiation source. Scanning electron microscopy (SEM) images were acquired using a Hitachi SU9000 field-emission scanning electron microscope and a Jeol JSM–IT100LA microscope equipped with an energy-dispersive X-ray spectroscopy (EDS) apparatus. UV–visible absorption and diffuse-reflectance spectra were recorded using spectrophotometers (V-670 and V-565, JASCO). Steady-state emission spectra were measured at room temperature under an Ar atmosphere using and spectrofluorometer (Fluorolog-3-21, Horiba and FP-8600, Jasco). N_2 and CO_2 adsorption measurements were conducted using a BELSORP-MAX II (MicrotracBEL) apparatus at liquid-N_2 temperatures (77 K) and 298 K, respectively. The samples were heated at 373 K for 1 h under reduced pressure before adsorption measurement. ^{13}C nuclear magnetic resonance (^{13}C NMR) spectra were acquired using a JEOL ECA400II (400 MHz) NMR spectrometer. XPS spectra were acquired using an ESCA-3400 X-ray photoelectron spectrometer (Shimadzu). The binding energies were calibrated by referencing the C 1s peak (285.0 eV) for each sample.

X-ray absorption fine structure (XAFS) measurements were conducted on the BL12C beamline of the Photon Factory at KEK (Inter-University Research Institute Corporation High Energy Accelerator Research Organization; Proposal No. 2020G597) using an electron energy of 2.5 GeV with an average current of 450 mA. The X-ray absorption spectra were acquired in transmission mode at room temperature using a Si(111) two-crystal monochromator. A pair of Ni-coated mirrors was used to eliminate higher harmonics. The X-ray absorption near-edge structure (XANES) spectra and extended X-ray absorption fine structure (EXAFS) oscillation were analyzed using the Athena software package.[2]

Photocatalytic Reactions. Reactions were performed at room temperature using an 8 mL test tube that contained 4 mg of catalyst powder and 4 mL of DMA solution containing 1.0 mM $[Ru(bpy)_3]Cl_2$ and 0.1 M BNAH. Prior to irradiation, the suspension was purged with CO_2 (Taiyo Nippon Sanso, >99.995%) for 30 min. A 400 W high-pressure Hg lamp (SEN lights Co.) was used as a light source, in combination with an aqueous $NaNO_2$ solution to allow for visible-light irradiation (λ > 400 nm). The formate generated in the liquid phase was analyzed using a capillary electrophoresis system (Otsuka Electronics, Agilent 7000). The gaseous reaction products were analyzed using a gas chromatograph equipped with a thermal conductivity detector (TCD) (GL Science, model GC323) and an activated carbon column; Ar was used as the carrier gas.

To investigate the effect of residual HCOOH in the as-prepared *α*-FeOOH/Al_2O_3, the powder sample (4 mg) was stirred in a 0.1 M NaOH solution (4 mL) for 3 h in the dark, and the supernatant solution was examined by capillary electrophoresis. The results showed that the supernatant contained 0.06 ± 0.008 μmol of HCOOH, which is much smaller than the amount produced by the photocatalytic reaction using *α*-FeOOH/Al_2O_3.

The turnover number (TON) for HCOOH production was calculated as

$$\text{TON} = \text{Amount of products} / \text{Amount of Fe atom in the catalyst.} \quad (1)$$

The apparent quantum yield (AQY) for HCOOH formation was measured using a 300 W Xe lamp (Asahi Spectra, MAX-303) fitted with a band-pass filter (460 nm) and was estimated as

$$\text{AQY}(\%) = (2 \times R/I) \times 100, \quad (2)$$

where *R* and *I* represent the rates of HCOOH production and incident photons, respectively. The total number of incident photons (12.1 mW) was measured using a spectroradiometer (Eko Instruments, LS-100). For the measurement, 8 mg of the *α*-

$FeOOH$(10.0 wt%)/Al_2O_3 catalyst (or bulk α-FeOOH) was suspended in 8 mL of DMA solution containing 1.0 mM $[Ru(bpy)_3]Cl_2$ and 0.1 M BNAH in a top-irradiation-type cell.

Selectivity toward HCOOH production during the CO_2 reduction reaction was calculated on the basis of the ratio between the amount of HCOOH generated and the total amount of reduction products (i.e., HCOOH, CO, and H_2):

$$\text{Selectivity to HCOOH / \%} = \text{HCOOH produced / Reduction products} \times 100, \qquad (3)$$

Isotope Tracer Experiment. $^{13}CO_2$ (^{13}C 99%, Watari CO., Ltd.) was purchased from Sigma-Aldrich. The $^{13}CO_2$ gas was introduced into a DMA solution (2 mL) containing 1.0 mM $[Ru(bpy)_3](PF_6)_2$ and 0.1 M BNAH, along with 4 mg of photocatalyst powder, after the liquids were degassed through freeze–pump–thaw cycling. After the samples were irradiated, the gas phase was analyzed using a gas chromatograph–mass spectrometer (Shimadzu, QP-2010-Ultra) equipped with a Molsieve5A capillary column. Prior to the measurement, no contamination of ^{13}CO and $H^{13}COOH$ in the $^{13}CO_2$ gas was detected. The liquid phase was analyzed by ^{13}C NMR. Before NMR analysis, the reacted solution was mixed with 0.1 M NaOH–D_2O solution in a volume ratio of 1:1. The solution was further diluted with 2 mL D_2O. After mixing, the solution was filtered using a micropore filter and the filtrate was used for NMR measurement. CD_3CN (>99.9%, Kanto Chemical) was used as an internal standard at 118.3 ppm.

A reference solution was prepared by mixing HCOOH, $[Ru(bpy)_3]Cl_2\cdot 6H_2O$, and BNAH in DMA at concentrations of 0.20 mM, 1.0 mM, and 0.1 M, respectively. The solution was then mixed with 0.1 M NaOH–D_2O solution in a volume ratio of 1:1.

Results and Discussion

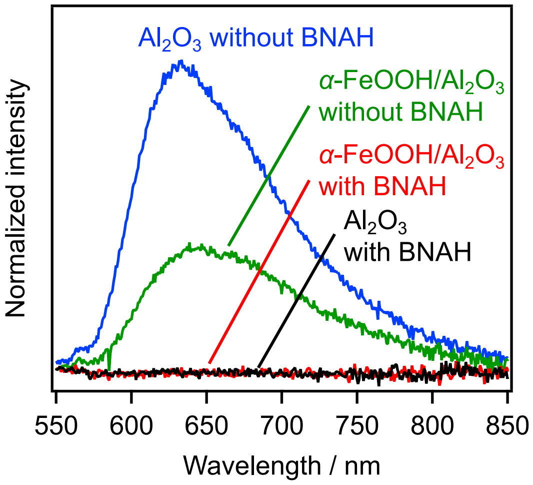

Figure S1. Steady-state emission spectra of $[Ru(bpy)_3]^{2+}$ in DMA solution containing catalysts and/or BNAH at room temperature. The excitation wavelength was 532 nm.

As shown in Figure S1, a clear emission peak was observed at around 640 nm, which originates largely from the lowest ^{3}MLCT excited state of $[Ru(bpy)_3]^{2+}$. In the presence of Al_2O_3, the emission quantum yield of $[Ru(bpy)_3]^{2+}$ without BNAH was 9.4%. When *α*-FeOOH/Al_2O_3 existed, the emission quantum yield was 4.4%, which was lower than that obtained with Al_2O_3. This is probably because *α*-FeOOH absorbs the excitation light (532 nm), thereby decreasing the number of photons that are supposed to be absorbed by $[Ru(bpy)_3]^{2+}$. This also makes it difficult to precisely measure the emission quantum yield. Adding BNAH in the solution resulted in a very low emission quantum yield of ~0.1% in both systems. The apparent quenching efficiency, which is defined by the ratio of the emission quantum yield with BNAH to that of without BNAH, was 99 and 98% for Al_2O_3 and *α*-FeOOH/Al_2O_3, respectively. The values are very close to the quenching efficiency of $[Ru(bpy)_3]^{2+}$ measured in a DMA/BNAH mixed solution (98%).[3] Summarizing above results, it is concluded that almost all $[Ru(bpy)_3]^{2+}$ are quenched reductively by BNAH and the one-electron-reduced species of $[Ru(bpy)_3]^{2+}$ donates an electron to the catalysts.

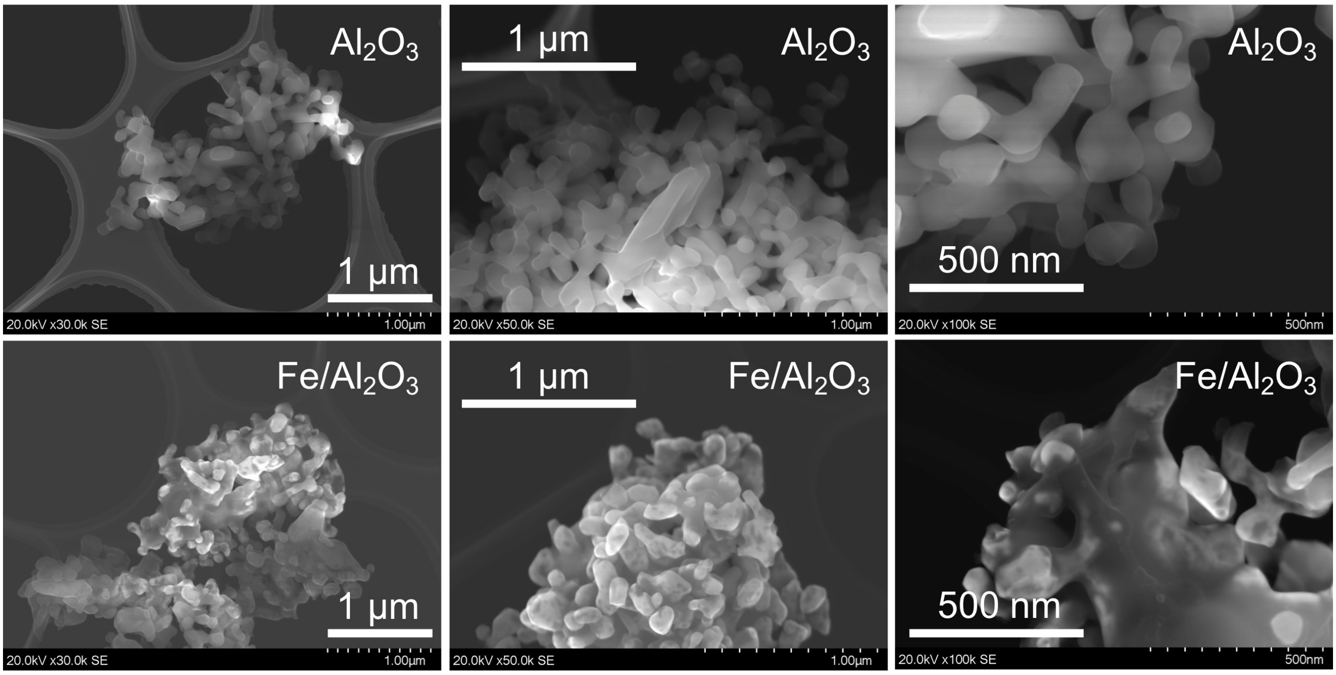

Figure S2. SEM images of 10.0 wt% Fe-loaded Al_2O_3 and unloaded Al_2O_3.

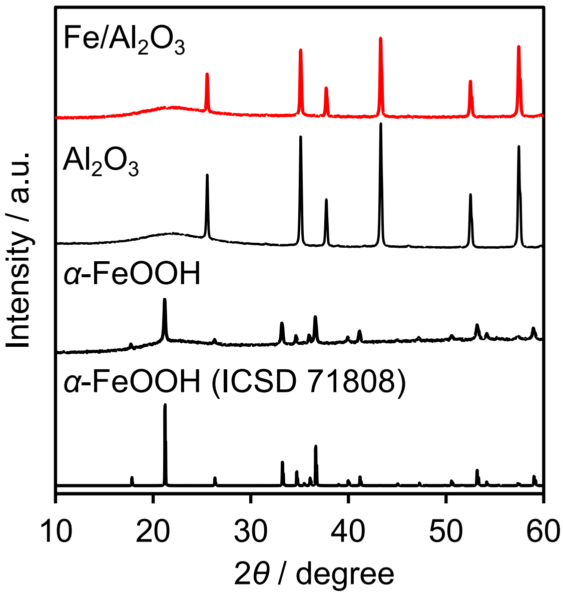

Figure S3. XRD patterns of 10.0 wt% Fe-loaded Al_2O_3, unloaded Al_2O_3, and the commercial *α*-FeOOH. A reference of *α*-FeOOH is also included.

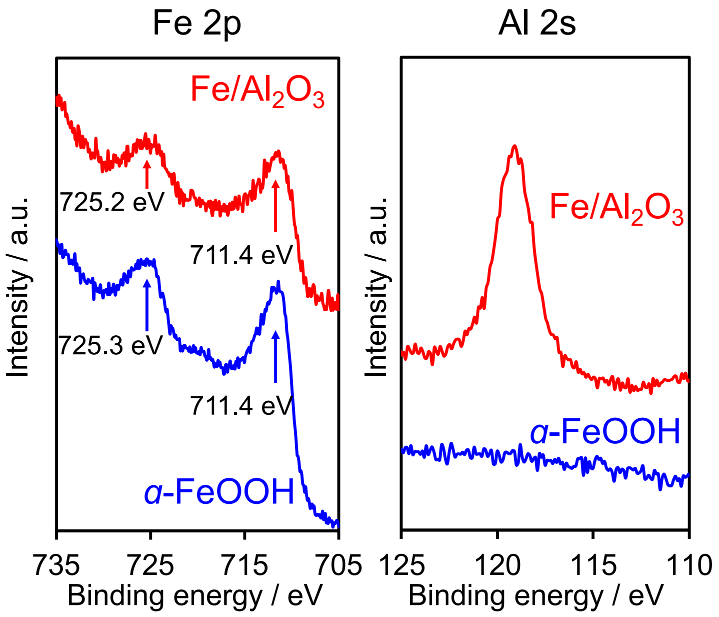

Figure S4. Fe 2p and Al 2s XPS spectra of 10.0 wt% Fe-loaded Al_2O_3 and bulk *α*-FeOOH.

As displayed in Figure S4, the catalyst sample shows two peaks at 711.4 and 725.2 eV, which are from Fe $2p_{3/2}$ and for Fe $2p_{1/2}$ photoelectrons, respectively. The peak positions are consistent with those reported previously.[4] The peak position of the catalyst sample in the Al 2s XPS (119.0 eV) is very close to that of Al_2O_3 (118.9 eV).[5]

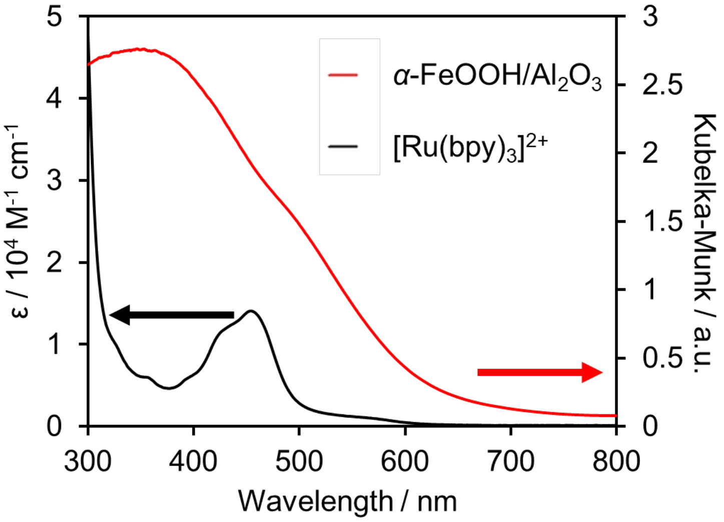

Figure S5. UV–visible absorption spectrum of $[Ru(bpy)_3]Cl_2{\cdot}6H_2O$ in DMA and the diffuse-reflectance spectrum of the 10.0 wt% *α*-$FeOOH/Al_2O_3$.

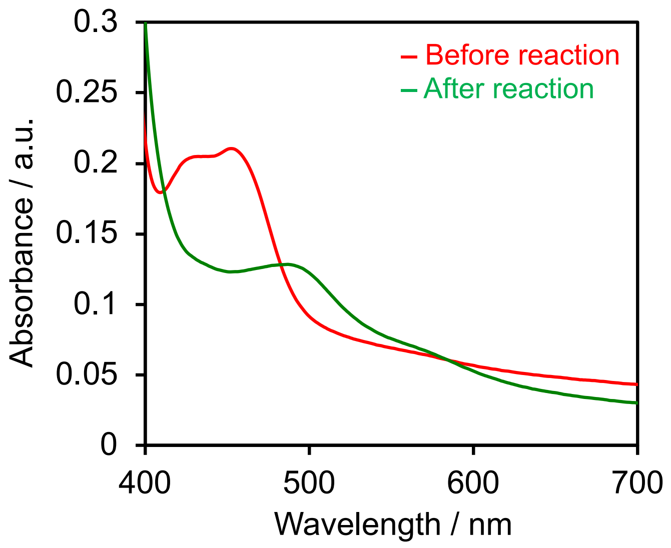

Figure S6. UV-visible absorption spectrum of the reaction solution after CO_2 reduction (3 h). The suspended solids were removed by filtration before measurement, and the supernatant solution was diluted by 100 times using DMA.

As shown in Figure S6, the solution before reaction exhibits a clear ^{1}MLCT absorption band at 400–500 nm region that is typical of **Ru**, although an additional absorption extending to longer wavelengths, which arises from the absorption of BNAH,[6] appears. The ^{1}MLCT absorption band of **Ru** became weaker and shifted to longer wavelengths after the reaction. This spectral change before and after the reaction is attributable to photochemical ligand substitution of the Ru(II) photosensitizer unit giving the corresponding Ru(II) bisdiimine-type complex(es).[7] The reversible deactivation of the *α*-$FeOOH/Al_2O_3$ system during the CO_2 reduction (Figure 2b) could therefore be explained in terms of the decomposition and/or structural change of **Ru**.

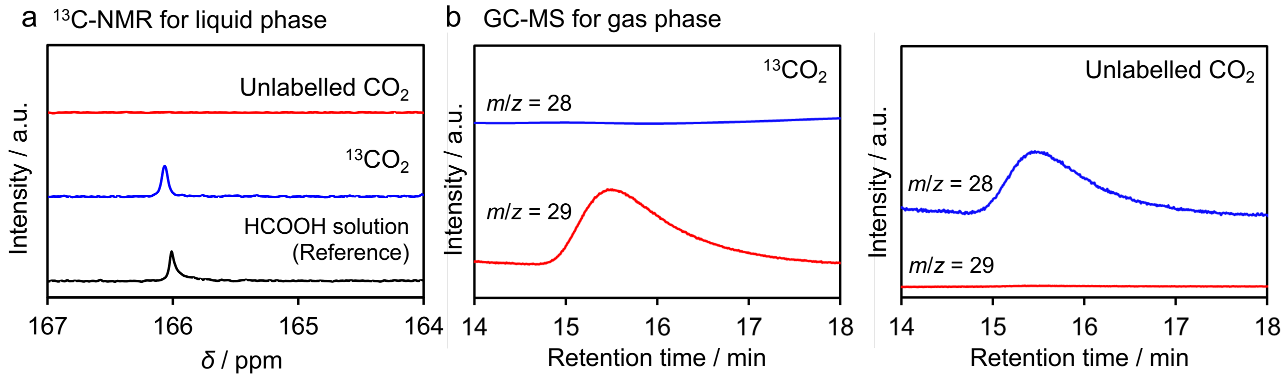

Figure S7. (a) ^{13}C NMR spectra of a 2 mL DMA solution containing **Ru (**1.0 mM**)**, BNAH (0.1 M), and 10 mg of α-FeOOH/Al_2O_3, which was measured after filtration. Data for HCOOH reference is also shown. A clear peak at δ = 166.1 ppm is assignable to $H^{13}COOH$.[8] (b) Gas chromatograms of the gas-phase products, as obtained using a mass spectrometer as a detector (*m/z* 28 and 29). The photocatalyst suspension was subject to visible-light irradiation from a 400 W high-pressure Hg lamp with a $NaNO_2$ solution filter for 15 h under $^{13}CO_2$ (610 Torr) and under saturated, unlabeled CO_2.

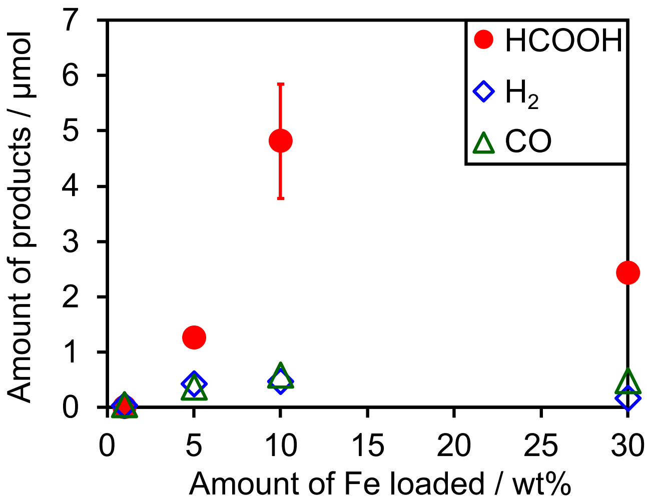

Figure S8. Dependence of HCOOH generation over α-FeOOH/Al_2O_3 on the loading amount of Fe. Reaction conditions: catalyst, 4 mg; reactant solution, a mixed solution of DMA containing $[Ru(bpy)_3]Cl_2$ (1.0 mM), BNAH (0.1 M); light source, a 400 W high-pressure Hg lamp with a $NaNO_2$ aqueous filter; reaction time, 3 h.

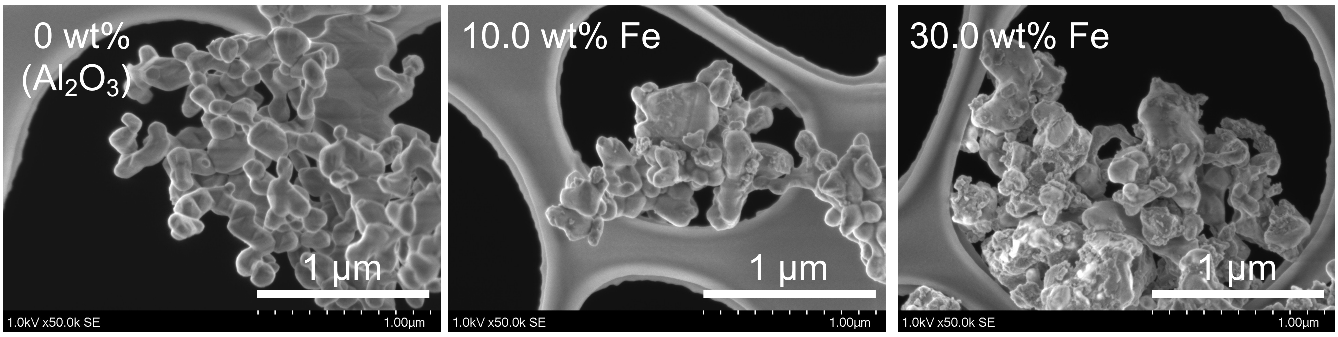

Figure S9. SEM images of Fe-loaded Al_2O_3 catalysts with different Fe loadings. It is obvious that roughening of the Al_2O_3 surface was more pronounced with increasing the loading amount of Fe, causing aggregation of the loaded Fe species at higher loadings.

Additional note on the result of AQY measurement. We have also measured AQY of the bulk *α*-FeOOH system in the same manner. The result showed that this system gave an AQY of 1.5% at 460 nm, which is approximately one-third of the *α*-FeOOH/Al_2O_3 system. In other words, the conversion efficiency of the incident photons at 460 nm is higher in the *α*-FeOOH/Al_2O_3 system than in the bulk. However, this seems to contradict the results (Table 1), which were obtained under 400 W high-pressure Hg lamp irradiation (λ > 400 nm); the bulk *α*-FeOOH system showed comparable activity than the *α*-FeOOH/Al_2O_3 system. As already mentioned, the *α*-FeOOH component does not work as a photocatalyst, even though it has a semiconductor-like absorption band (see Figure S5). Therefore, the *α*-FeOOH component should cause a light-filtering effect to different extents depending on the concentration of *α*-FeOOH. A plausible explanation for the higher AQY of the *α*-FeOOH/Al_2O_3 system is that the light filtering effect under 460 nm irradiation is less pronounced in the *α*-FeOOH/Al_2O_3 system, as compared to the bulk *α*-FeOOH system. This may be another advantage of the *α*-FeOOH/Al_2O_3 system over the bulk system.

References

[1] D. Mauzerall, F. H. Westheimer, *J. Am. Chem. Soc.* **1955**, *77*, 2261-2264.
[2] B. Ravel, M. Newville, *J. Synchrotron Radiat.* **2005**, *12*, 537-541.
[3] Y. Kuramochi, M. Kamiya, H. Ishida, *Inorg. Chem.* **2014**, *53*, 3326-3332.
[4] J. Baltrusaitis, D. M. Cwiertny, V. H. Grassian, *Phys. Chem. Chem. Phys.* **2007**, *9*, 5542-5554.
[5] G. E. McGuire, G. K. Schweitzer, T. A. Carlson, *Inorg. Chem.* **1973**, *12*, 2450-2453.
[6] N. W. Gaikwad, L. Yang, E. G. Rogan, E. L. Cavalieri, *Free Radic. Biol. Med.* **2009**, *46*, 253-262.
[7] P. E. Hoggard, G. B. Porter, *J. Am. Chem. Soc.* **1978**, *100*, 1457-1463.
[8] N. R. Babij, E. O. McCusker, G. T. Whiteker, B. Canturk, N. Choy, L. C. Creemer, C. V. D. Amicis, N. M. Hewlett, P. L. Johnson, J. A. Knobelsdorf, F. Li, B. A. Lorsbach, B. M. Nugent, S. J. Ryan, M. R. Smith, Q. Yang, *Org. Process Res. Dev.* **2016**, *20*, 661-667.

Author Contributions

D.A.: most experiments and writing of the manuscript. S.N.: catalyst preparation, emission measurements, photocatalytic reaction, discussion and conceptualization. S.Y.: SEM/EDS measurements. T.K.: XAFS measurements. Y.K.: CO_2 adsorption, NMR measurements and discussion. T.Y.: SEM/EDS measurements. S.N.: XAFS measurements. K.M.: conceptualization, funding acquisition, and supervision. All authors contributed to the final version of the manuscript.
